# Participation in the community eligibility provision – a universal free school meals policy in the United States in 2014–2020

**DOI:** 10.1057/s41271-025-00590-w

**Published:** 2025-09-01

**Authors:** Anna M. Localio, Melissa A. Knox, Paul L. Hebert, Jennifer Sonney, Tom Lindman, Jessica C. Jones-Smith

**Affiliations:** 1 Department of Health Systems and Population Health, University of Washington, School of Public Health, 3980 15 Ave NE, Seattle, WA 98195 USA; 2 Department of Economics, University of Washington, Seattle, WA USA; 3 Health Services Research and Development Center of Innovation for Veteran-Centered and Value-Driven Care, Veterans Affairs Puget Sound Health Care System, Seattle, WA USA; 4 Department of Child, Family, and Population Health Nursing, University of Washington School of Nursing, Seattle, WA USA; 6 Center for Population and Development Studies, Harvard University, Cambridge, MA USA; 5 School of Public Policy and Governance, University of Washington Evans, Seattle, WA USA; 7 Department of Health, Society and Behavior Joe C. Wen School of Population Health, University of California, Irvine, CA USA

**Keywords:** School meals, Nutrition policy, Policy implementation, Federal policy, School health

## Abstract

We assessed the association of participation in the Community Eligibility Provision, a universal free school meals policy in the United States, with school and area-level characteristics, and how these associations changed between 2014 and 2020. Using logistic regression models with district-clustered standard errors, in 53,391 eligible schools nationwide, adjusted prevalence of participation was 3.8 percentage points (pp) lower among high schools relative to elementary schools (95% CI: 1.6, 6.0), 8.9 pp lower in small relative to large school districts (95% CI: 4.8, 13.0), 10.9 pp lower in suburban relative to urban schools (95% CI: 6.9, 14.8), and 13.3 pp lower in schools in non-Medicaid expansion relative to expansion states (95% CI: 17.1, 20.7). Over time prevalence of participation in majority Hispanic schools increased to a lesser extent than participation in majority Black schools. Addressing barriers to policy adoption in schools with persistently lower participation will be critical to increase equitable access to universal free school meals.

**Supplementary Information:**

The online version contains supplementary material available at https://doi.org/10.1057/s41271-025-00590-w.

## Key messages

- Schools with younger students, with higher student economic need, and with a majority of Black students were positively associated with participation in the Community Eligibility Provision.
- Schools in larger school districts, in rural regions, and in areas with high poverty were positively associated with participation in the Community Eligibility Provision.
- Schools in Medicaid expansion states and those in the Southeastern and Northern US were positively associated with participation in the Community Eligibility Provision.

## Introduction

Eligibility for the Community Eligibility Provision (CEP), a federal universal free school meals (UFM) policy for low-income schools, expanded to millions of additional schools in the United States (US) in fall 2023. The policy, which already served over 40,000 schools and 20 million children in 2023 [1] now has the potential to reach even more students. More schools than ever are interested in CEP after many temporarily experienced UFM during the COVID-19 pandemic, when the US Department of Agriculture (USDA) issued nationwide waivers. However, questions remain regarding whether the policy is accessible to all eligible schools interested in participating.

Nearly all meals served in US schools are provided through the USDA’s National School Lunch Program (NSLP) and School Breakfast Program (SBP) [2]. Children from qualifying low-income households receive school meals for free or reduced price; others pay a price set by the local school district [2]. Most meals are served for free or reduced price [2]. For students to qualify for free or reduced-price meals, households must either apply by certifying their income eligibility, or students are directly certified by states or school districts for free meals without the need for household applications [3]. Students are identified for direct certification if their household participates in a federal assistance program like the Supplemental Nutrition Assistance Program (SNAP). Students identified as homeless, migrant, or in some states, as participating in Medicaid, are also directly certified for free meals. In 2010, the Healthy, Hunger-Free Kids Act established CEP, allowing schools and school districts in qualifying low-income communities to decide to opt in to this non-pricing school meals program, enabling them to provide free breakfast and lunch to all students [4]. To be eligible for CEP, schools must have an identified student percentage (ISP) of 40% or greater. ISP is calculated using the proportion of directly certified students in a school. USDA reimburses based on a formula that results in schools with an ISP of 62.5% and above receiving as much as they would if all meals had been certified as free. Schools with ISP between 40 and 62.5% need to cover any shortfall from other funds such as additional funding from their district or state.

CEP increases participation in school meals through SBP and NSLP [5], which offer better dietary quality than alternatives and reduce disparities in diet quality across income and race and ethnicity groups [5–8]. This is particularly true after the Healthy, Hunger-Free Kids Act improved nutrition standards, requiring increased fruits, vegetables, whole grains, and low-fat milk and decreased sodium present in meals [6]. CEP reduces the stigma associated with receiving free meals by extending free meals to all students, rather than just those from low-income households and eliminates the need for applications. It also extends access to children whose household falls above the income eligibility threshold ($40,560 annually for a family of four as of 202) [11]. CEP is associated with improvements in student academic, behavioral, and health outcomes, as well as increased disposable income for low-income families [5, 12–14].

Several studies have examined school- and community-level factors associated with the decision to opt into CEP participation. Turner and colleagues assessed schools in California between 2013 and 2017 using repeated cross-sectional analyses, finding that CEP participation was more common among schools that were larger, predominantly Hispanic, and rural [15]. Rogus and colleagues assessed school districts cross-sectionally in school year 2015–16 in a sample representing approximately 60% of all districts nationwide, finding that ISP was positively associated with CEP participation, and that this association varied by district size [16]. They also found that districts were more likely to participate in the Southeast and in rural areas [16]. Hecht and colleagues conducted a cross-sectional analysis of schools nationwide in school year 2017–18 and found differences in participation by length of state CEP availability and school ISP, Title I participation, locale, level, enrollment, size, student race/ethnicity, and geographic region [17]. Authors consistently found that schools in larger districts with a greater share of low-income students were more likely to opt into CEP [15–17]. However, other findings were conflicting; for example, school-level analyses found inconsistent relationships of CEP with school size, level, and rurality [15, 17], which could have implications for how resources are allocated and where outreach efforts are focused.

The purpose of this study was to assess the overall association of CEP participation with school- and area-level characteristics among CEP-eligible schools and estimate how these associations have changed over time.

## Data and methods

### Conceptual framework

Selection of characteristics (described under “Independent Variables” below) was based on existing evidence and guided by the Consolidated Framework for Implementation Research (CFIR). CFIR provides a menu of constructs associated with effective implementation in order to identify barriers and facilitators to implementation [18]. Constructs are organized into five domains: innovation (policy being implemented), outer setting (broader contextual environment in which the policy is implemented), inner setting (specific setting where the policy is implemented), individuals (characteristics of individuals who implement, receive, or otherwise influence/are influenced by the intervention), and implementation process (activities and strategies used to implement the innovation) [18]. Characteristics, units of measure, and underlying constructs they are hypothesized to measure are displayed in Table 1.

**Table 1:** Variables hypothesized to be associated with CEP adoption using the Consolidated Framework for Implementation Research (CFIR)

**Construct**	**Construct Definition**	**Measures:**	**Rationale:**
Innovation Cost	Degree to which the innovation purchase and operating costs are affordable	School ISP^a^	Costs are a major concern for schools when implementing school meal programs [37]. We hypothesize that cost is a central barrier to CEP participation. Therefore, we included four measures of policy cost.
		School Size	CEP adoption is most affordable for schools with ISP above 62.5%—the threshold at which all meals are reimbursed at the free rate, which is associated with CEP adoption [16, 17].
		District Size	Larger schools and/or districts may benefit from economies of scale, for whom the policy may be more affordable [15–17], 24;
		US Region	Food and labor costs vary by region, which could impact program affordability [16, 17, 24]
Local Attitudes	Degree to which sociocultural values and beliefs encourage the Outer Setting to support implementation and/or delivery of the innovation	School Locale	Local attitudes differ by urbanicity which may impact policy adoption
		State Medicaid Expansion	Schools in states that have expanded Medicaid may be more amenable to adopting other safety net programs [17]
Local Conditions	Degree to which economic, environmental, political, and/or technological conditions enable the Outer Setting to support implementation and/or delivery of the innovation	School Locale	Rural areas experience higher poverty relative to urban are as [29], which could drive CEP adoption [15].
		Zip Code Level Neighborhood Deprivation	Schools in high-poverty areas may be incentivized to adopt CEP to address higher rates of food insecurity
Partnerships & Connections	Degree to which the Inner Setting is networked with external entities	US Region	Outreach and support to schools and districts occurs at the regional level and may impact CEP participation rates [16, 17, 23]
Policies & Laws	Degree to which legislation, regulations, etc. support implementation and/or delivery of the innovation	State Medicaid Expansion	Medicaid expansion increases direct certification in some states [4], which could increase school meal participation and incentivize CEP adoption
Need	Degree to which the individuals have deficits related to survival, well-being, or personal fulfillment, which will be addressed by the innovation	School ISP	ISP reflects student economic need; schools with higher ISP may adopt CEP to address unmet student nutrition needs [16, 17].
		School Majority Race and Ethnicity	Racially and ethnically minoritized students are more likely to experience food insecurity [33]; schools with more minoritized students may be driven to adopt CEP to address need.
		Zip Code Level Neighborhood Deprivation	;Neighborhood deprivation may reflect student need beyond ISP, which could drive adoption
Innovation Recipients	Individuals who are directly or indirectly receiving the innovation	School Level	School meal participation rates are higher among elementary school students [26], which may drive adoption.
		School Majority Race and Ethnicity	Meal participation rates are higher among Black and Hispanic compared to White students [26], which may drive adoption.

Table includes study measures hypothesized to be associated with CEP adoption, their data source, and the rationale for the hypothesized association. The measures are organized according to the Consolidated Framework for Implementation Research, which includes five domains (innovation, outer setting, inner setting, individuals, and implementation process). Selected constructs and their definition are included under selected domains. Variables were assigned to the underlying construct they are intended to measure. Some variables capture more than one construct

### Data sources

School CEP eligibility and participation data were obtained from state departments of education, NCES, Food Research and Action Committee (FRAC) [4], and replication data from another CEP researcher [14, 19]. Details on data acquisition processes for school data are included in Supplementary Material Part 1**.** School-level characteristics and locale were obtained from the National Center for Education Statistics (NCES) [19, 20]. Area-level characteristics were obtained from Robert Graham Center [21], Medicaid expansion status from KFF (formerly the Kaiser Family Foundation) [22], and regions from USDA [23].

### Study population, dependent and independent variables

The analysis included all public and charter schools nationwide that participated in CEP in any year during the study period. Schools with an ISP of 40% or higher, (used as a proxy for school CEP eligibility) but that did not participate in CEP during the study period were also included as a comparison group. The dependent variable was school-level CEP participation (binary). Participation data, were measured yearly between 2014 and 2020. Independent variables included school- and area-level characteristics hypothesized to be associated with CEP participation, organized by CFIR constructs, as shown in Table 1. Some measures capture more than one construct. We selected a total of 11 variables. School-level variables include ISP (categorized into deciles), percent Black, Hispanic, and White students (the largest race and ethnicity groups represented), enrollment (categorized as fewer than 400 students; 400–699; 700–99; 1000 or more), and school level (elementary; middle; high; other, like K-12). Community-level variables include school district size (fewer than 10 school; 10–20 schools; 21–30 schools; greater than 30 schools), school locale (urban; suburban; town; rural, constructed from census tracts and blocks based on population density) [20], and neighborhood deprivation measured by the Social Deprivation Index (SDI), a composite score with a scale from 0 to 100 with 100 indicating the highest deprivation [21]. We utilized this index because it was available for all years of this analysis, it was measured at the zip code level, and it is a well-validated measure of neighborhood deprivation [21]. Regional characteristics include state Medicaid expansion status (as binary variable) and USDA region (Western; Mountain Plains; Southwest; Midwest; Mid-Atlantic; Northeast; Southeast). Table 1 describes rationale for selection of independent variables. We categorized some variables to be consistent with the published literature [15, 17].

### Statistical analysis

Data analysis occurred between November 2023 and April 2024. Data were assessed for missingness which ranged from 0 to 2% among all variables except ISP, for which 19% of observations were missing. To address missingness, we used multiple imputation by chained equations. We used two logistic regression models mutually adjusted for all independent variables as well as year, treated as a continuous variable. Standard errors were clustered at the school district level. The model to assess the overall association of CEP participation with school- and area-level characteristics did not include interactions. A second model included interactions with time for all characteristics to assess time trends in these associations. Details of these models are included in Supplementary Material Part 2. For ease of interpretability, from both models we computed predicted probabilities, multiplied by 100 to obtain the estimated prevalence of CEP participation. For continuous variables, to interpret results, we computed change in prevalence of participation for specific values. For percent race and ethnicity variables, we computed change in prevalence of participation for an average school comprised of a majority (75%) of each race or ethnicity group, setting the other race or ethnicity variables to 10% each so that these proportions did not add up to more than 100%. For SDI, we modeled change in participation for schools in zip codes with an SDI score at the 25th, 50th, and 75th percentiles of the sample, which correspond to scores of 52, 72, and 87, respectively. We performed Wald tests for all categorical variables to test the null hypothesis that coefficients for all category levels were equal to zero. To assess whether results differed by district-level CEP adoption, as a secondary analysis, we stratified schools by whether they were in a partially adopting versus a fully adopting or fully non-adopting school district. The secondary analysis excluded schools in districts with only one school because these districts did not have a choice to be fully verus partially adopting. We conducted all analyses using StataMP 18.

## Results

Table 2 displays characteristics by school year of 53,391 distinct schools eligible for CEP between 2014 and 2020. Not all schools report measurements in all years, as some opened or closed during this period. Table 2 also displays number of distinct schools contributing measurements in each year. Time-varying characteristics remained stable except for schools in Medicaid expansion states, which increased during the study period as more states expanded Medicaid, and ISP category, which fluctuated across years. Depending on the year, 21–29% of schools reported an ISP less than 40%. However, because CEP eligibility is redetermined every four years, most of these schools remained eligible to participate.

**Table 2:** Characteristics of schools eligible for the Community Eligibility Provision by school year

	2014–2015	2015–2016	2016–2017	2017–2018	2018–2019	2019–2020
Total Unique Schools = 53,391	N Schools = 49,168	N Schools = 49,165	N Schools = 48,922	N Schools = 48,898	N Schools = 48,588	N Schools = 47,941
School-Level Characteristics						
School Level (N (%))						
Elementary	29,239 (59%)	29,181 (59%)	29,148 (60%)	29,271 (60%)	29,184 (60%)	28,995 (60%)
Middle/Jr. High	7551 (15%)	7,490 (15%)	7,502 (15%)	7,519 (15%)	7,454 (15%)	7,362 (15%)
High	7745 (16%)	7,906 (16%)	7,802 (16%)	7,809 (16%)	7,844 (16%)	7,610 (16%)
Other (e.g., K-12)	4633 (9%)	4,588 (9%)	4,471 (9%)	4,299 (9%)	4,107 (8%)	3,975 (8%)
School Enrollment (N (%))						
Small (< 400 students)	22,476 (46%)	22,604 (46%)	22,670 (46%)	22,793 (47%)	22,857 (47%)	22,739 (47%)
Medium (400–699 students)	17,443 (35%)	17,459 (36%)	17,273 (35%)	17,277 (35%)	17,135 (35%)	16,777 (35%)
Large (700–999 students)	6,087 (12%)	5,949 (12%)	5,859 (12%)	5,742 (12%)	5,533 (11%)	5,344 (11%)
Very Large (≥ 1000 students)	3,162 (6%)	3,152 (6%)	3,120 (6%)	3,087 (6%)	3,063 (6%)	3,082 (6%)
Identified Student Percentage (ISP) (N (%))						
Less than 40%	12,035 (24%)	10,868 (22%)	13,947 (29%)	10,030 (21%)	10,683 (22%)	12,585 (26%)
40–50%	15,486 (31%)	15,352 (31%)	12,403 (25%)	12,152 (25%)	12,014 (25%)	11,960 (25%)
50–60%	8,921 (18%)	9,638 (20%)	8,779 (18%)	10,094 (21%)	9,830 (20%)	9,152 (19%)
60–70%	6,695 (14%)	7,397 (15%)	6,963 (14%)	8,839 (18%)	8,344 (17%)	7,240 (15%)
70–80%	3,603 (7%)	3,569 (7%)	3,940 (8%)	4,464 (9%)	4,587 (9%)	4,138 (9%)
80–90%	1,663 (3%)	1,630 (3%)	2,014 (4%)	2,383 (5%)	2,369 (5%)	2,058 (4%)
90–100%	765 (2%)	711 (1%)	877 (2%)	935 (2%)	762 (2%)	808 (2%)
Percent Students by Race and Ethnicity (mean (sd))						
White	38% (0.15%)	38% (0.15%)	37% (0.15%)	36% (0.15%)	36% (0.15%)	35% (0.15%)
Hispanic	30% (0.14%)	31% (0.14%)	31% (0.14%)	32% (0.14%)	32% (0.14%)	33% (0.14%)
Black	24% (0.13%)	23% (0.13%)	23% (0.13%)	23% (0.13%)	23% (0.13%)	22% (0.13%)
Asian	3% (0.03%)	3% (0.03%)	3% (0.03%)	3% (0.03%)	3% (0.03%)	3% (0.03%)
Native American or Alaska Native	2% (0.05%)	2% (0.05%)	3% (0.06%)	3% (0.06%)	3% (0.05%)	3% (0.06%)
Pacific Islander or Native Hawaiian	0.3% (0.01%)	0.3% (0.01%)	0.3% (0.01%)	0.3% (0.01%)	0.3% (0.01%)	0.3% (0.01%)
Multiple Races	3% (0.02%)	3% (0.02%)	3% (0.02%)	3% (0.02%)	4% (0.02%)	4% (0.02%)
Area-Level Characteristics						
School District Size (N (%))						
Small (≤ 10 schools)	19,906 (40%)	20,040 (41%)	19,839 (41%)	19,715 (40%)	20,142 (41%)	19,903 (42%)
Medium (11–20 schools)	7,140 (15%)	7,080 (14%)	7,132 (15%)	7,180 (15%)	6,997 (14%)	6,921 (14%)
Large (21–30 schools)	4,294 (9%)	4,278 (9%)	4,275 (9%)	4,238 (9%)	4,126 (8%)	4,027 (8%)
Very Large (≥ 31 schools)	17,828 (36%)	17,767 (36%)	17,676 (36%)	17,765 (36%)	17,323 (36%)	17,090 (36%)
Urbanicity/Rurality (N (%))						
Urban	18,106 (37%)	18,125 (37%)	18,100 (37%)	18,090 (37%)	18,064 (37%)	17,903 (37%)
Suburban	11,959 (24%)	11,891 (24%)	11,799 (24%)	11,838 (24%)	11,768 (24%)	11,649 (24%)
Town	7,333 (15%)	7,380 (15%)	7,258 (15%)	7,193 (15%)	7,066 (15%)	6,877 (14%)
Rural	11,770 (24%)	11,768 (24%)	11,766 (24%)	11,777 (24%)	11,690 (24%)	11,512 (24%)
Social Deprivation Index (mean (sd))*	68 (10)	68 (10)	68 (10)	68 (10)	68 (10)	68 (11)
Medicaid Expansion State (N (%))	26,233 (53%)	27,212 (55%)	28,345 (58%)	28,329 (58%)	29,127 (60%)	29,049 (61%)
USDA Region (N(%))						
Western	7731 (16%)	7,750 (16%)	7,687 (16%)	7,699 (16%)	7,822 (16%)	7,774 (16%)
Southwest	10,674 (22%)	10,693 (22%)	10,674 (22%)	10,648 (22%)	10,686 (22%)	10,560 (22%)
Mountain Plains	2717 (6%)	2,733 (6%)	2,749 (6%)	2745 (6%)	2,709 (6%)	2,653 (6%)
Midwest	8330 (17%)	8,283 (17%)	8,294 (17%)	8357 (17%)	8,348 (17%)	8,261 (17%)
Southeast	10,656 (22%)	10,626 (22%)	10,534 (22%)	10,586 (22%)	10,355 (21%)	10,106 (21%)
Mid-Atlantic	4054 (8%)	4,033 (8%)	4,013 (8%)	3982 (8%)	3,786 (8%)	3,722 (8%)
Northeast	5006 (10%)	5,047 (10%)	4,971 (10%)	4881 (10%)	4,882 (10%)	4,865 (10%)

Table includes characteristics for 53,391 unique public schools nationwide that were ever eligible for CEP between 2014 and 2020. Social Deprivation Index score is a composite measure of area-level deprivation ranging from 1 to 100 with 100 corresponding to highest level of deprivation [21] Counts may not sum precisely to column totals due to multiply imputed data and rounding; discrepancies are ≤ 1 school and do not affect reported percentages

**Fig. 1:**
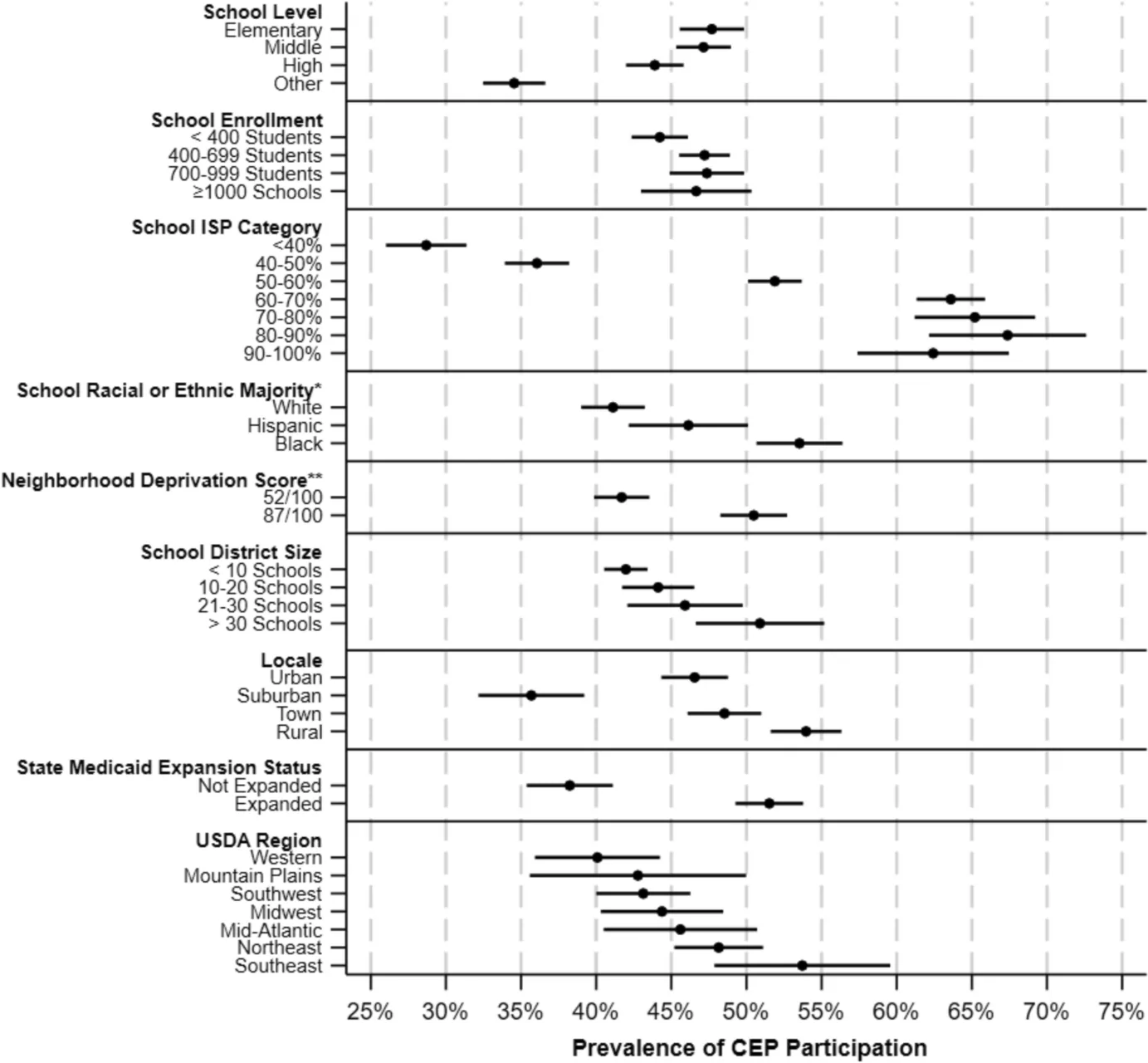
Adjusted associations of school and area characteristics with the prevalence of participation in the Community Eligibility Provision. Figure shows point estimates and 95% confidence intervals of prevalence of CEP participation by school and area characteristics adjusted for covariates for 53,391 schools eligible for CEP between 2014 and 2020. Standard errors were clustered at the district level. Results of joint tests for all categorical variables that all categories are equal to zero were statistically significant at the *p* < .001 level. *Each race or ethnicity variable (White, Hispanic, Black) was set to 75% while the other two were each simultaneously set to 10% **Scores represent the 25th percentile and 75th percentile of the distribution of the Social Deprivation Index [21]

Figure 1 displays adjusted estimates and 95% confidence intervals of overall prevalence of CEP participation by school and area characteristics. Supplementary Material Table S1 shows both unadjusted and adjusted prevalence point estimates and confidence intervals, and Table S2 shows these in terms of average marginal effects. Elementary schools had the highest prevalence of participation (3.80 percentage points (pp) higher than high schools (95% CI: 1.64 pp, 5.95 pp)). School size was associated with participation; schools with 700–999 students had a 3.13 pp higher prevalence of participation than schools with fewer than 400 students (95% CI: 5.25 pp, 1.02 pp). ISP was positively associated with participation; relative to schools with ISP of 40–50%, schools with an ISP of 50–60% had a 15.84 pp higher prevalence of participation (95% CI: 26.33 pp, 36.13 pp) and schools with an ISP of 60–70% had a 27.54 pp higher prevalence of participation (95% CI: 24.96 pp, 30.13 pp). By school race and ethnicity demographics, prevalence of participation was 3.59 pp lower for each 25-pp increase in percent White students (95% CI: −5.11 pp, −2.07 pp).

School districts with more than 30 schools had a higher prevalence of CEP participation than districts with 20 or fewer schools (8.92 pp higher for the largest districts relative to the smallest, 95% CI: 4.81, 13.04 pp). A 25-point increase in neighborhood deprivation score was associated with a 5.99 pp higher prevalence of participation (95% CI: 4.89, 7.09 pp). By locale, suburban schools had the lowest prevalence of participation (10.87 pp lower than urban schools, 95% CI: -14.79, -6.94 pp) and rural schools had the highest (7.41 pp higher than urban schools, 95% CI: 4.51, 10.32 pp). State Medicaid expansion was associated with a 13.28 pp higher prevalence of participation relative to non-expansion states (95% CI: 9.53, 17.03 pp). By region, schools in the Southeast had the highest overall prevalence of participation (13.64 pp higher than Western states, 95% CI: 5.87, 21.40 pp) followed by schools in the Northeast (8.07 pp higher than Western states, 95% CI: 3.28, 12.87 pp). Results of joint tests for all categorical variables that all categories were equal to zero were statistically significant (p < 0.05), indicating that variables were associated overall with CEP participation.

Supplementary Material Table S3 depicts the results of the secondary analysis stratified by whether schools were in a partially adopting school district. There were 27,142 schools in districts that partially adopted CEP and 20,569 schools in districts that fully adopted or fully did not adopt CEP. Partially adopting school districts had a larger mean number of schools (83) than districts that fully adopted (16 schools) or fully did not adopt (13 schools). In both strata, the relationship with CEP participation was similar to overall estimates by ISP, locale, neighborhood deprivation, and Medicaid expansion status. However, results of the stratified analyses differed from the overall analysis by school level, school size, race and ethnicity, district size, and region. While higher school level remained negatively associated with CEP participation among schools in partially adopting districts, among schools in fully adopting districts, high schools had a 4.72 pp higher prevalence of participation compared to elementary schools (95% CI: 3.10, 6.34 pp). While larger school size was associated with higher CEP participation in the overall analysis, only medium-sized schools were associated with higher participation than small schools among schools in partially adopting districts. School size was not significantly associated with CEP participation among schools in fully adopting districts. By race and ethnicity, percent White students was not significantly associated with CEP participation in schools in partially adopting districts as it was in the overall analysis. However, both percent White students and percent Hispanic students were negatively associated with participation in schools in fully adopting districts.

While very large districts were associated with higher prevalence of CEP participation in the overall analysis, among schools in fully adopting districts, those in very large districts had a 14.01 pp lower prevalence of participation than schools in very small districts (95% CI: −22.85, −6.17). In contrast, there was no statistically significant relationship between district size and CEP participation among schools in partially adopting districts. By region, compared to the West, among schools in fully adopting districts, those in the Southeast and Northeast had higher prevalence of participation, similar to findings in the overall analysis. However, among schools in fully adopting districts, those in the Mid-Atlantic, Southwest, and Mountain Plains also had a higher prevalence of participation compared to schools in the West. Among schools in partially adopting districts on the other hand, only those located in the Midwest and Mid-Atlantic had higher prevalence of participation than those in the West.

**Fig. 2:**
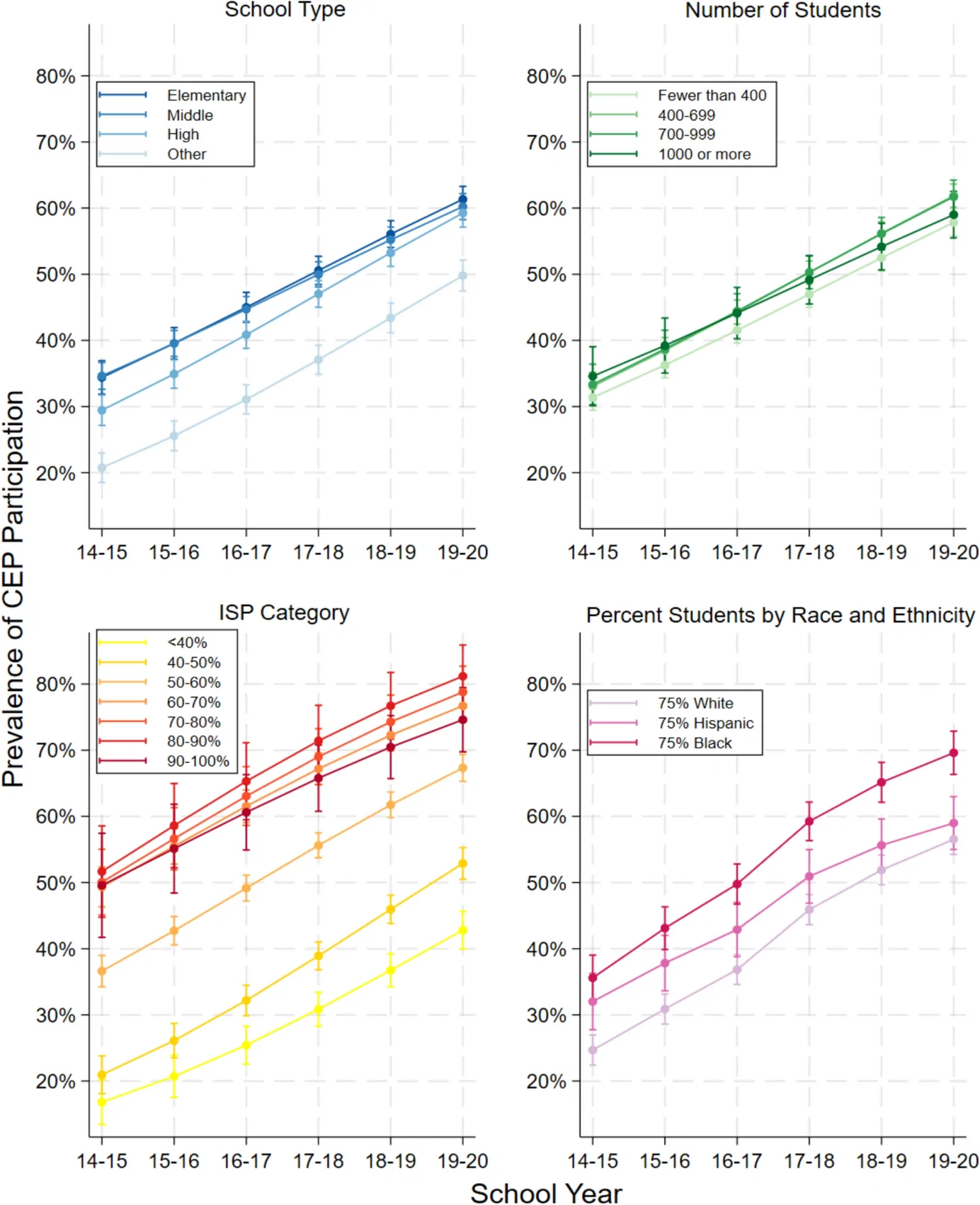
Adjusted associations of school-level characteristics with annual prevalence of participation in the Community Eligibility Provision. Figures show prevalence of CEP participation for 53,391 schools eligible for CEP between 2014 and 2020, by year and category, adjusted for covariates. Standard errors were clustered at the district level. ISP is the school identified student percentage. For school level, “other” represents a combination of primary and secondary school grades not captured by elementary, middle, or high (e.g., K-12). For each graph, all covariates are set to their means. For graph of percent students by race or ethnicity, each race/ethnicity variable (White, Hispanic, Black) was set to 75% while the other two were each simultaneously set to 10%

**Fig. 3:**
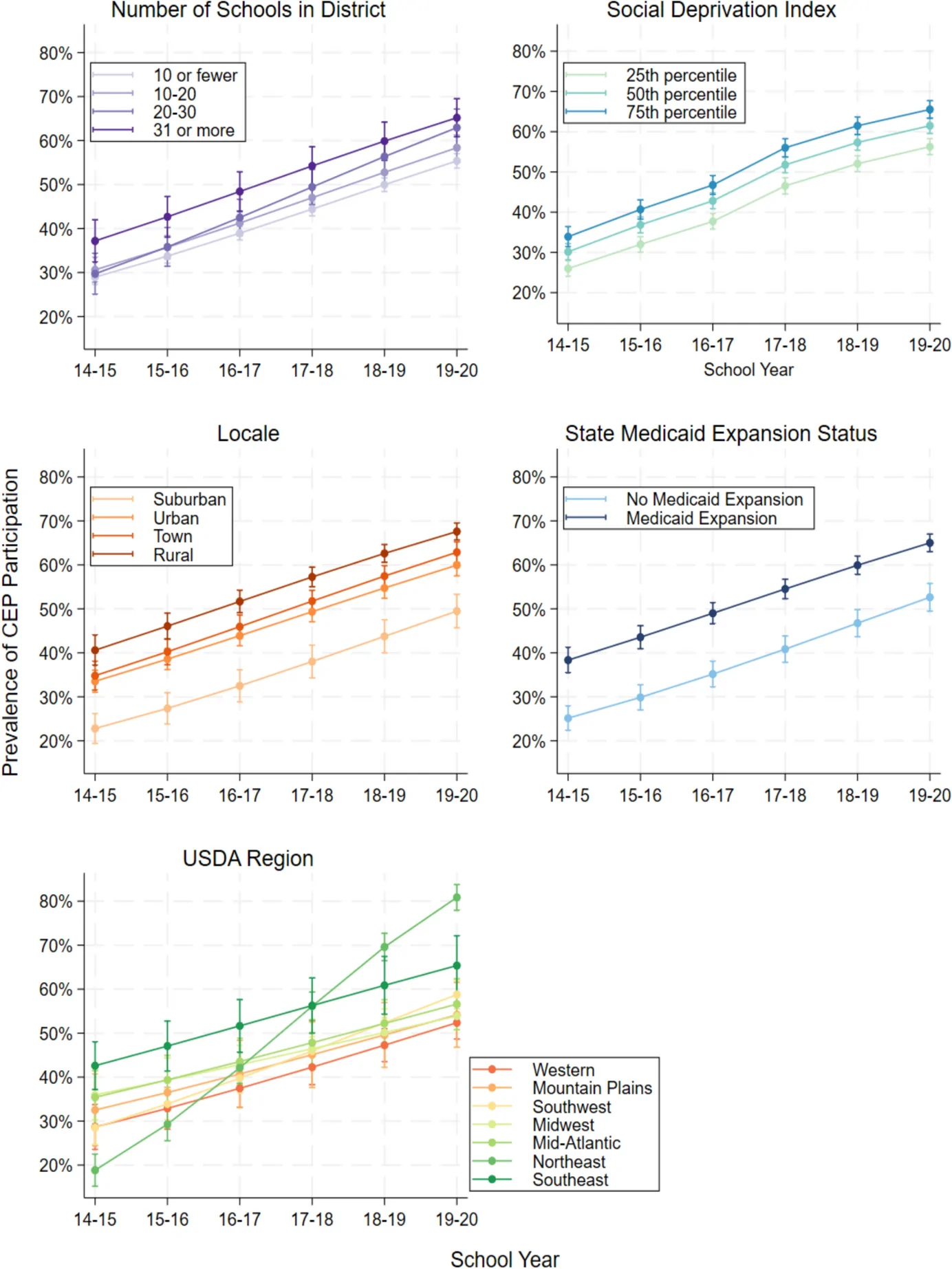
Adjusted associations of area-level characteristics with annual prevalence of participation in the Community Eligibility Provision. Figures show prevalence of CEP participation for 53,391 schools eligible for CEP between 2014 and 2020, by year and category, adjusted for covariates Standard errors were clustered at the district level. For Social Deprivation Index, the 25th percentile corresponds to a score of 52/100, 50th percentile to a score of 72/100, and 75th percentile to a score of 87/100

Trends in prevalence of CEP participation over time are shown by school (Fig. 2) and area characteristics (Fig. 3). Supplementary Material Figures S1 and S2 display these trends modeled with year as a categorical (rather than continuous) variable. Table S4 includes point estimates and confidence intervals of average annual change in log-odds of participation. Between 2014 and 2020, overall change in prevalence of participation was higher in high schools (29.82 pp, 95% CI: 27.35 pp, 32.28 pp) than elementary schools (26.93 pp, 95% CI: 24.99 pp, 28.88 pp). Among schools with a majority of Black students, change in prevalence of participation was 31.81 pp (95% CI: 27.83 pp, 35.79 pp), vs. 24.85 pp for schools with a majority of Hispanic students (95% CI: 21.65 pp, 28.06 pp). By region, change in prevalence was highest in the Northeast (62.03 pp, 95% CI: 57.16 pp, 66.89 pp, vs. 23.72 pp in the West, 95% CI: 19.20 pp, 28.26 pp).

## Discussion

In this study of all CEP-eligible schools nationwide, we examined longitudinal trends in school CEP participation between 2014 and 2020 and assessed the degree to which characteristics of CEP-participating schools changed over time. These trends in CEP participation reveal potential barriers and facilitators to participation, both persistent and emerging. At the school level, we found lower adjusted prevalence of CEP participation in high schools compared to elementary schools and in schools with ISP below 50% compared to schools with higher ISPs. We also observed lower participation in schools with predominantly Hispanic and predominantly White students compared to those with predominantly Black students. Additionally, small schools and districts had lower participation compared to their larger counterparts. By locale, we observed that suburban schools had the lowest participation while rural schools had the highest. Schools in non-Medicaid expansion states had lower participation than those in states that expanded Medicaid. Regionally, schools in the Mountain Plains and Western US had the lowest participation while schools in the Northeast and Southeast had the highest.

Our findings were mostly consistent with those from previous research [15–17] but sometimes differed. Specifically, Hecht and colleagues found associations of CEP participation with school size, school level, and locale that conflicted with ours (though ours were consistent with those from Turner and colleagues [15]). However, other takeaways such as associations of participation with larger school districts and higher percent Black students were consistent with our findings [17]. Hecht and colleagues included more variables in their analysis, [17] while we attempted to limit the number of variables in our model capturing a single construct. Additionally, the estimates reported by Hecht et al. are from school year 2017–18 only, [17] whereas ours are from school year 2014–15 through 2019–20, and some associations changed over time.

As we hypothesized using the Consolidated Framework for Implementation Research, many observed differences in CEP participation are likely driven by affordability. For CEP-participating schools, the percent of meals reimbursed at the federal free rate is determined by multiplying ISP by 1.6 (an approximation of students certified via household application). Schools with an ISP of 62.5% or higher are reimbursed at the free rate for 100% of meals served. For eligible schools that fall below this break-even threshold (approximately 70% of schools in the sample), factors influencing affordability may play an important role in the decision to participate in CEP. For example, participation was relatively high in the Southeast where food costs are lower, while areas like the Mountain Plains and West where food costs are higher were associated with lower prevalence of participation [24]. However, other factors, such as varying levels of outreach by regional USDA offices, as posited by Hecht and colleagues, may also contribute to this difference [17]. Similarly, lower participation among small schools and districts likely reflects higher per-meal costs than larger schools and districts which can take advantage of economies of scale [24].

Trends in participation over time revealed schools with ISP over 60% adopted CEP earlier, likely because 62.5% is the threshold at which all meals served are reimbursed at the free rate. Relatively lower participation persisting over time among schools with an ISP between 40 and 50% likely indicates that many schools in this category, though eligible, feel unable to afford to participate in CEP because they would still be responsible for the costs of 20–36% of meals served not reimbursed by the federal government. Although the threshold for CEP eligibility was lowered in fall 2023 from an ISP of 40% to 25%, without changing the multiplier that determines the federal reimbursement rate, it is unlikely that such eligibility expansions will result in substantial increases in CEP participation unless CEP funds are augmented by state or local support. The USDA included funding for this change in their 2025 budget proposal [25].

Other differences in CEP participation may be attributed to factors such as student demand. Elementary schools were positively associated with CEP participation and appear to have adopted the policy earlier than middle, high, or other school levels. This is likely driven by higher school meal participation [26]. However, the gap in CEP participation between elementary and high schools lessened over time as more high schools adopted the policy. High schools have historically had lower school meal participation [26], possibly due to lower satisfaction with school meals [27], despite improvements in quality [6]. However, CEP adoption increases school meal participation [5]. Particularly for students who eat lunch off campus, switching to school meals could have additional benefits like reduced absenteeism and improved nutrition, but school meal acceptability could remain a barrier in this age group. This is an area for future research.

Observed differences by locale may also be explained by student demand and/or need. We observed that rural schools had the highest prevalence of CEP participation of any locale. Previous research has shown that rural schools have higher school meal participation prior to CEP adoption [28]. The finding that CEP participation is high in rural areas is promising since poverty is higher in rural compared to urban areas [29]. Although rural areas face higher food transportation costs, use of food purchasing cooperatives may facilitate adoption [24, 30]. Suburban schools lagged behind urban and rural schools in participation. Poverty in the suburbs is increasing at a faster rate than in other locales, yet the suburban social safety net remains less developed [31]. Lower exposure and access to safety net programs in suburban areas may exacerbate stigma and decrease willingness to adopt a program like CEP [32]. Need also likely explains the positive association of CEP participation with neighborhood deprivation. That neighborhood deprivation predicts school CEP participation independently of school ISP indicates that the policy is responsive to measures of area-level need beyond the proportion of households participating in means-tested safety net programs (i.e., ISP).

Differences in student need may also explain observed differences in CEP participation by race and ethnicity. Schools with predominantly Black students were associated with a higher change in prevalence of participation than predominantly Hispanic schools, and this gap in participation widened over time. On the one hand, the finding that CEP is reaching schools serving Black students is promising since Black students face higher risk of food insecurity than their White counterparts due to structural racism [33], and CEP addresses food insecurity [5]. However, Hispanic children are also at an increased risk of food insecurity [33], yet participation was comparatively lower among schools serving Hispanic students. Research is needed to determine barriers to adoption for schools serving this population.

A non-cost-related facilitator of CEP participation that likely contributed to observed regional differences may be partnerships and connections. For example, high prevalence of participation in early years in the Southeastern US may have been facilitated by earlier rollout of the policy in this region, and thus earlier establishment of regional networks [34]. Schools in the Northeast had a higher change in prevalence of participation than any other region during this period. This may be due to the concerted effort of a large coalition of stakeholders to implement CEP in all New York City public schools by 2017 [35]. Such strategies could be modified to increase participation in other regions, such as suburban areas.

Finally, we found evidence that state-level amenability to social safety net policies may facilitate CEP participation, as schools in Medicaid expansion states were associated with participation. This is an important factor for policy adoption to consider moving forward. As of 2025, several states including California, Maine, Colorado, Minnesota, and New Mexico have implemented UFM for all public schools, pledging to cover the difference in meal costs and federal reimbursement using state funding. Future research should assess whether, and to what extent, eliminating this cost barrier has allowed for successful implementation of UFM statewide. Although such federal-state partnerships represent a promising approach to implementing UFM, they are likely to be limited to those states amenable to social safety net policies. For example, of the 10 states that have not expanded Medicaid as of 2025, none have implemented state UFM policies [36]. For non-UFM states, CEP represents the most viable mechanism for schools to provide free, nutritious meals for low-income children. Therefore, addressing barriers to CEP participation remains critical.

Stratified analyses revealed that associations also differed by whether schools were in a partially adopting school district. However, because partially adopting districts were on average much larger than fully adopting or fully non-adopting districts, this may have influenced differences in decision making. For example, district size was inversely associated with CEP participation in fully adopting districts compared to results of the main analysis. This may be because it was more difficult for larger districts to achieve universal CEP adoption than for districts with fewer schools. We also found that in fully adopting districts, high schools had a higher prevalence of CEP participation compared to elementary schools, which was opposite of our overall findings. It is possible that district-level adoption could facilitate CEP participation among high schools. Finally, strong positive associations with CEP participation among schools in fully adopting districts in the Northeastern and Southeastern US indicate that these regions may have put forth a concerted effort for CEP adoption at the district level.

### Limitations

This study has several limitations. Reported estimates can only be interpreted as associations as this study was not designed to determine causal relationships. Although our selection of variables was guided by CFIR and existing literature, we may not have captured important determinants of CEP adoption such as other political dimensions or measures of institutional capacity. There is also likely heterogeneity in adoption that this study design did not capture. Finally, given the structure of CEP participation data sources, we treated all schools as if they remained participating after adoption. However, up to 6% of schools in the sample de-adopted the policy, so participation may be slightly overestimated.

## Conclusion

Between 2014 and 2020, each year thousands of eligible schools adopted the Community Eligibility Provision, a federal universal free school meals policy. This analysis of characteristics associated with school CEP participation found that schools with younger students, with higher student economic need, and with a majority of Black students were positively associated with CEP participation. Additionally, schools in larger school districts, in rural regions, and in areas with high poverty were positively associated with CEP participation. Finally, CEP participation was associated with schools in Medicaid expansion states, and those in the Southeastern and Northeastern US. These associations suggest that schools likely make the participation decision based largely on their perceived ability to afford participation, but other factors also play a role. Possible facilitators to participation based on observed associations include partnerships and connections, student demand, and both student- and area-level need. As CEP and other UFM policies continue to expand, understanding barriers and facilitators to participation in these schools will be critical to successful policy implementation.

## Supplementary Information

Below is the link to the electronic supplementary material.

Supplementary file1 (DOCX 389 KB)

## Data Availability

Data are available from the corresponding author upon reasonable request.

## References

[1] Food Research and Action Center (FRAC). Community eligibility: the key to hunger-free schools, School Year 2022–2023. Published online May 2023. Accessed December 8, 2023. https://frac.org/wp-content/uploads/cep-report-2023.pdf

[2] USDA Economic Research Service. National School Lunch Program. Published online November 25, 2024. Accessed December 31, 2024. https://www.ers.usda.gov/topics/food-nutrition-assistance/child-nutrition-programs/national-school-lunch-program/

[3] Moore Q, Conway K, Kyler B, Gothro A. *Direct Certification in the National School Lunch Program: State Implementation Progress, School Year 2012–2013*. US Department of Agriculture, Food and Nutrition Service, Office of Policy Support; 2013.

[4] Food Research & Action Center. Community Eligibility. Accessed August 15, 2024. https://frac.org/community-eligibility

[5] Cohen JFW, Hecht AA, McLoughlin GM, Turner L, Schwartz MB. Universal school meals and associations with student participation, attendance, academic performance, diet quality, food security, and body mass index: a systematic review. Nutrients. 2021;13(3):911. 10.3390/nu13030911. PMC8000006 33799780

[6] Kinderknecht K, Harris C, Jones-Smith J. Association of the Healthy, Hunger-Free Kids Act with dietary quality among children in the US National School Lunch Program. JAMA. 2020;324(4):359. 10.1001/jama.2020.9517. PMC7388023 32721008

[7] Smith TA, Lin BH, Guthrie J. School meal nutrition standards reduce disparities across income and race/ethnicity. Am J Prev Med. 2024;67(2):249–57. 10.1016/j.amepre.2024.03.012. 38521131

[8] Liu J, Micha R, Li Y, Mozaffarian D. Trends in food sources and diet quality among US children and adults, 2003–2018. JAMA Netw Open. 2021;4(4):e215262–e215262. 10.1001/jamanetworkopen.2021.5262. PMC8042524 33844000

[9] Bhatia R, Jones P, Reicker Z. Competitive foods, discrimination, and participation in the National School Lunch Program. Am J Public Health. 2011;101(8):1380–6. 10.2105/AJPH.2011.300134. PMC3134491 21680939

[10] Zuercher MD, Cohen JFW, Hecht CE, Hecht K, Ritchie LD, Gosliner W. Providing school meals to all students free of charge during the COVID-19 pandemic and beyond: challenges and benefits reported by school foodservice professionals in California. Nutrients. 2022;14(18):3855. 10.3390/nu14183855. PMC9500887 36145229

[11] US Department of Agriculture Food and Nutrition Service. Child Nutrition Programs: Income Eligibility Guidelines (2023–2024). Published online February 9, 2023. Accessed January 3, 2024. https://www.fns.usda.gov/cn/fr-020923

[12] Marcus M, Yewell KG. The effect of free school meals on household food purchases: evidence from the Community Eligibility Provision. J Health Econ. 2022;84:102646. 10.1016/j.jhealeco.2022.102646. 35792362

[13] Localio AM, Knox MA, Basu A, Lindman T, Walkinshaw LP, Jones-Smith JC. Universal free school meals policy and childhood obesity. Pediatrics. 2024. 10.1542/peds.2023-063749. PMC10979297 38495019

[14] Ruffini K. Universal access to free school meals and student achievement: evidence from the community eligibility provision. J Human Resour. 2022;57(3):776–820. 10.3368/jhr.57.3.0518-9509R3.

[15] Turner L, Guthrie JF, Ralston K. Community eligibility and other provisions for universal free meals at school: impact on student breakfast and lunch participation in California public schools. Transl Behav Med. 2019;9(5):931–41. 10.1093/tbm/ibz090. 31328770

[16] Stephanie Rogus, Joanne Guthrie, Katherine Ralston. Characteristics of School Districts Offering Free School Meals to All Students Through the Community Eligibility Provision of the National School Lunch Program. U.S. Department of Agriculture, Economic Research Service; 2018.

[17] Hecht AA, Stuart EA, Pollack Porter KM. Factors associated with universal free school meal provision adoption among US public schools. J Acad Nutr Diet. 2022;122(1):49–63. 10.1016/j.jand.2021.06.282. 34427189

[18] Damschroder LJ, Reardon CM, Widerquist MAO, Lowery J. The updated consolidated framework for implementation research based on user feedback. Implement Sci. 2022;17(1):75. 10.1186/s13012-022-01245-0. PMC9617234 36309746

[19] National Center for Education Statistics. Common Core of Data. Accessed December 29, 2023. https://nces.ed.gov/ccd/

[20] Geverdt D. Education Demographic and Geographic Estimates Program (EDGE): Locale Boundaries User’s Manual. Published online December 2015. Accessed April 23, 2025. https://nces.ed.gov/programs/edge/Geographic/LocaleBoundaries

[21] Robert Graham Center. Social Deprivation Index (SDI). Accessed October 23, 2023. https://www.graham-center.org/maps-data-tools/social-deprivation-index.html

[22] KFF. Status of State Medicaid Expansion Decisions. 2023. Accessed December 29, 2023. https://www.kff.org/medicaid/issue-brief/status-of-state-medicaid-expansion-decisions-interactive-map/

[23] Food and Nutrition Service, U.S. Department of Agriculture. FNS Regional Offices. Published online 2023. Accessed December 29, 2023. https://www.fns.usda.gov/fns-regional-offices

[24] Michael Ollinger, Joanne Guthrie, Audrey Peo. USDA School Meal Programs: how and why the cost of food purchases varies across locales. Published online November 2018. Accessed December 8, 2023. https://files.eric.ed.gov/fulltext/ED605439.pdf

[25] US Department of Agriculture. 2025 Budget Summary. Published online 2024. Accessed August 23, 2024. https://www.usda.gov/sites/default/files/documents/2025-usda-budget-summary.pdf

[26] US Department of Agriculture Food and Nutrition Service, Office of Policy Support. *School Nutrition and Meal Cost Study, Final Report Volume 4: Student Participation, Satisfaction, Plate Waste, and Dietary Intakes*.; 2019. https://fns-prod.azureedge.us/sites/default/files/resource-files/SNMCS-Volume4.pdf

[27] O’Donnell AJ, Mansfield D, Loiacono B, et al. Barriers and facilitators to high school meal program participation: a qualitative study of students and their caregivers. J Sch Health. 2022;92(12):1186–93. 10.1111/josh.13229. 35878882

[28] Rothbart MW, Schwartz AE, Gutierrez E. Paying for free lunch: the impact of CEP universal free meals on revenues, spending, and student health. Educ Finance Policy. 2023. 10.1162/edfp_a_00380.

[29] US Department of Agriculture Economic Research Service. Rural Poverty & Well-Being. Published online November 15, 2023. Accessed December 31, 2023. https://www.ers.usda.gov/topics/rural-economy-population/rural-poverty-well-being/

[30] Michael Ollinger, Joanne Guthrie. *Economies of Scale, the Lunch-Breakfast Ratio, and the Cost of USDA School Breakfasts and Lunches*. United States Department of Agriculture; 2015.

[31] Allard SW. *Places in Need*. Russell Sage Foundation; 2017. Accessed February 26, 2024. http://www.jstor.org/stable/10.7758/9781610448659

[32] Allard SW, Pelletier E. Volatility and change in suburban nonprofit safety nets. RSF. 2023;9(2):134–60. 10.7758/rsf.2023.9.2.06. PMC11088929 38737982

[33] Odoms-Young A, Bruce MA. Examining the impact of structural racism on food insecurity: implications for addressing racial/ethnic disparities. Fam Community Health. 2018;41(Suppl 2 Suppl):S3–6. 10.1097/FCH.0000000000000183. PMC5823283 29461310

[34] Nueberger Z, Segal B, Nchako C, Masterson K. Take Up of Community Eligibility This School Year. Center for Budget and Policy Priorities. February 25, 2015. Accessed August 19, 2024. https://www.cbpp.org/research/take-up-of-community-eligibility-this-school-year

[35] Watts A, Araiza AL, Fernández CR, et al. The campaign for universal free lunch in new york city: lessons learned. Pediatrics. 2021;148(4):e2020049734. 10.1542/peds.2020-049734. 34526351

[36] Marissa Sheldon. States that Have Passed Universal Free School Meals (So Far). Hunter College New York City Food Policy Center. April 9, 2024. Accessed August 23, 2024. https://www.nycfoodpolicy.org/states-with-universal-free-school-meals-so-far-update

[37] Ralston K, Newman C, Clauson A, Guthrie J, Buzby J. The National School Lunch Program: Background, Trends, and Issues. Economic Research Service, US Department of Agriculture; 2008.

