# Supplementary material for: Participation in the community eligibility provision – a universal free school meals policy in the United States in 2014–2020
Source: J Public Health Policy. 2025 Sep 1;46(4):795–815. doi: 10.1057/s41271-025-00590-w (PMC13694218; doi:10.1057/s41271-025-00590-w)
Supplement: Supplementary file 1 [file 41271_2025_590_MOESM1_ESM.docx]

*Original article*

**Participation in the Community Eligibility Provision -- a Universal Free School Meals Policy in the United States in 2014-2020**

Anna M. Localio*, PhD, MPH^1^, Melissa A. Knox, PhD^2^, Paul L. Hebert, PhD^1,3^, Jennifer Sonney, PhD, APRN, PPCNP-BC^4^, Tom Lindman, MPP^5,6^, Jessica C. Jones-Smith, PhD, MPH, RD^1,7^

^1^Department of Health Systems and Population Health, University of Washington School of Public Health, Seattle, WA, USA

^2^Department of Economics, University of Washington, Seattle, WA, USA

^3^ Health Services Research & Development Center of Innovation for Veteran-Centered and Value-Driven Care, Veterans Affairs Puget Sound Health Care System, Seattle, WA, USA

^4^Department of Child, Family, and Population Health Nursing, University of Washington School of Nursing, Seattle, WA, USA

^5^University of Washington Evans School of Public Policy & Governance, Seattle, WA, USA

^6^Center for Population and Development Studies, Harvard University, Cambridge, MA, USA

^7^Department of Health, Society and Behavior, Joe C. Wen School of Population Health, University of California, Irvine, CA, USA

**Supplementary Material**

**Part 1.** Data acquisition process

We requested CEP eligibility (measured via the identified student percentage or ISP) and participation data directly via email from state departments of education who maintained the most complete and detailed records of any data source we encountered. We obtained complete data from most states, however some states did not maintain records. Therefore, we used National Center for Education Statistics (NCES) Common Core of Data to fill in remaining missing data.^1^ NCES reports CEP participation status for most schools nationwide. Because NCES does not report school ISP directly, we used the proportion of students reported as directly certified for free meals multiplied by 1.6 as a proxy for school ISP. We also obtained school and district demographic data from NCES. Because missing CEP data still remained, we used data obtained from the Food Research Action Center, who maintain a nationwide database of school CEP participation and ISP.^2^ However, these records were not complete in all years – particularly early years. Additionally, these records lacked school identifiers, requiring us to match on school name, including using fuzzy string matching in Stata when naming conventions differed slightly. Finally, because we still lacked CEP participation data for some states in early years, we used replication data from another CEP researcher^3^ to fill in remaining missing data. However, these data only spanned through 2017 and did not contain ISP.

**Part 2.** Statistical model equation and description

To assess the overall association of school and area-level characteristics with CEP participation, we fit the following logistic regression model (Model 1):

$logit\left( E\left[ Y_{ij}|X_{ij} \right] \right)= \beta_{0}+\beta_{1}{SchType}_{i}+{\beta_{2}{ISP}_{i}+ \beta_{3}{SchSize}_{i}+\beta_{4}{PercHisp}_{i}+\beta_{5}{PercBlack}_{i}+\beta_{6}{PercWhite}_{i}+\beta_{7}{DistSize}_{i}+\beta_{8}{SDI}_{i}+\beta_{9}{UrbRur}_{i}+\beta_{10}{MedicaidExp}_{i}+\beta_{11}{Region}_{i}+ \beta}_{12}{Year}_{j}$

Y_ij_ represents the log-odds of CEP participation for year *i* and school *j*, conditional on covariates. β's of independent variables (β_1_- β_11_) represent the association of characteristics with CEP participation, adjusted for covariates including time trends (β_12_). To assess time trends in these associations, we added interactions with time in years for all covariates (Model 2). In Model 2, β's of independent variables interacted with year represent the change in associations over time. Because participation increased approximately linearly over time, we specified year as a continuous variable in both models, but show results from our second model with year as an indicator variable in Appendix Figures A and B.

**Table S1**. Associations of school and area characteristics with prevalence of participation in the Community Eligibility Provision

|  | **Unadjusted Prevalence^a^** | **95% CI** | **Adjusted Prevalence** | **95% CI** |
| --- | --- | --- | --- | --- |
| School Level | | | | |
| Elementary | 48.62 | 45.97, 51.27 | 47.70 | 45.56, 49.84 |
| Middle/Jr. High | 45.30 | 43.07, 47.53 | 47.15 | 45.32, 48.97 |
| High | 42.28 | 40.08, 44.47 | 43.90 | 41.99, 45.82 |
| Other (e.g. K-12) | 34.36 | 32.05, 36.66 | 34.54 | 32.47, 36.61 |
| Enrollment | | | | |
| Small (<400 students) | 42.84 | 41.08, 44.60 | 44.23 | 42.36, 46.11 |
| Medium (400-699 students) | 49.38 | 46.81, 51.95 | 47.21 | 45.52, 48.90 |
| Large (700-999 students) | 48.60 | 44.67, 52.52 | 47.37 | 44.89, 49.85 |
| Very Large (≥ 1000 students) | 42.95 | 37.87, 48.03 | 46.66 | 42.98, 50.33 |
| ISP Category | | | | |
| <40% | 23.38 | 20.66, 26.09 | 28.69 | 26.01, 31.37 |
| 40-50% | 32.59 | 30.23, 34.96 | 36.06 | 33.92, 38.20 |
| 50-60% | 53.18 | 50.99, 55.36 | 51.89 | 50.11, 53.68 |
| 60-70% | 69.21 | 66.46, 71.95 | 63.60 | 61.33, 65.88 |
| 70-80% | 73.18 | 68.78, 77.59 | 65.20 | 61.19, 69.22 |
| 80-90% | 77.17 | 71.86, 82.47 | 67.34 | 62.15, 72.61 |
| 90-100% | 66.68 | 61.90, 71.47 | 62.43 | 57.39, 67.47 |
| School Race and Ethnicity Composition | | | | |
| 75% Percent White^b^ | 32.54 | 30.92, 34.16 | 41.12 | 39.00, 43.24 |
| 75% Percent Hispanic^c^ | 47.20 | 42.94, 51.46 | 46.14 | 42.17, 50.11 |
| 75% Percent Black^d^ | 66.93 | 62.98, 70.88 | 53.53 | 50.67, 56.39 |
| District Size | | | | |
| Small (≤ 10 schools) | 37.87 | 36,82, 38.91 | 41.98 | 40.54, 43.42 |
| Medium (11-20 schools) | 42.96 | 40.08, 45.84 | 44.13 | 41.72, 46.53 |
| Large (21-30 schools) | 44.41 | 39.90, 48.93 | 45.91 | 42.08, 49.75 |
| Very Large (≥ 31 schools) | 56.36 | 51.09, 61.64 | 50.90 | 46.63, 55.18 |
| Locale | | | | |
| Urban | 58.51 | 54.87, 62.16 | 46.55 | 44.34, 48.77 |
| Suburban | 35.24 | 31.93, 38.56 | 35.69 | 32.17, 39.21 |
| Town | 40.15 | 38.21, 42.10 | 48.54 | 46.08, 50.99 |
| Rural | 40.45 | 38.91, 41.98 | 53.97 | 51.62, 56.32 |
| SDI Score |  |  |  |  |
| 25^th^ percentile (52/100) | 35.30 | 33.55, 37.06 | 41.70 | 39.86, 43.54 |
| 75^th^ percentile (87/100) | 57.44 | 54.78, 60.10 | 50.48 | 48.26, 52.70 |
| Medicaid Expansion Status | | | | |
| Not Expanded | 42.59 | 38.97, 46.22 | 38.24 | 35.38, 41.11 |
| Expanded | 48.22 | 45.49, 50.95 | 51.52 | 49.27, 53.78 |
| USDA Region | | | | |
| Western | 40.86 | 34.75, 46.98 | 40.08 | 35.92, 44.24 |
| Mountain Plains | 32.23 | 25.61, 38.86 | 42.78 | 35.58, 49.98 |
| Southwest | 40.66 | 36.42, 44.90 | 43.13 | 40.00, 46.26 |
| Midwest | 44.51 | 37.79, 51.23 | 44.38 | 40.31, 48.45 |
| Mid-Atlantic | 49.16 | 41.60, 56.72 | 45.60 | 40.50, 50.71 |
| Northeast | 55.37 | 51.63, 59.12 | 48.15 | 45.20, 51.11 |
| Southeast | 53.60 | 47.48, 59.72 | 53.72 | 47.87, 59.57 |

Table shows prevalence of CEP participation by school and area characteristics adjusted for covariates for 53,391 schools eligible for CEP between 2014-2020. Results of joint tests for all categorical variables that all categories are equal to zero were statistically significant at the p<.001 level.

^a^Unadjusted models were separate logistic regression models, adjusted for year, and with standard errors accounting for clustering at the district level.

^b^Percent Hispanic and percent Black each set to 10%

^c^Percent Black and percent White each set to 10%

^d^Percent Hispanic and percent White each set to 10%

**Table S2.** Relative associations (marginal effects) of school and area characteristics with prevalence of participation in the Community Eligibility Provision

|  | **Unadjusted Average Marginal Effect^a^** | **95% CI** | **Adjusted Average Marginal Effect** | **95% Confidence Interval** |
| --- | --- | --- | --- | --- |
| **School-Level Characteristics** | | | | |
| Level (ref Elementary) | | | | |
| Middle/Jr. High | -3.32*** | -5.12, -1.52 | -0.55 | -2.03, 0.93 |
| High | -.6.34*** | -8.05, -4.63 | -3.80** | -5.95, -1.64 |
| Other (e.g. K-12) | -14.27*** | -17.00, -11.53 | -13.16*** | -15.21, -11.11 |
| Enrollment (ref Small: < 400 students)) | | | | |
| Medium (400-699 students) | 6.54*** | 4.78, 8.29 | 2.98*** | 1.80, 4.16 |
| Large (700-999 students) | 5.76** | 2.47, 9.04 | 3.13** | 1.02, 5.25 |
| Very Large (≥ 1000 students) | 0.11 | -4.26, 4.48 | 2.42 | -0.56, 5.405 |
| ISP Category (ref < 40%) | | | | |
| 40-50% | 9.22*** | 7.45, 10.98 | 7.37*** | 5.73, 9.00 |
| 50-60% | 29.80*** | 27.35, 32.25 | 23.20*** | 20.81, 25.60 |
| 60-70% | 45.83*** | 42.52, 49.14 | 34.91*** | 31.77, 38.05 |
| 70-80% | 49.81*** | 45.20, 54.41 | 36.51*** | 32.26, 40.76 |
| 80-90% | 53.79*** | 48.54, 59.04 | 38.69*** | 33.43, 43.94 |
| 90-100% | 43.31*** | 38.08, 48.53 | 33.74*** | .28.38, 39.09 |
| Race and Ethnicity (25-percentage point change) | | | | |
| Percent Hispanic | 0.79 | -0.87, 2.44 | -1.72 | -3.64, 0.19 |
| Percent White | -8.40*** | -9.66, -7.14 | -3.59*** | -5.11, -2.07 |
| Percent Black | 9.86*** | 8.50, 11.22 | 0.99 | -0.66, 2.64 |
| **Area-Level Characteristics** | | | | |
| School District Size (ref Small: ≤ 10 schools) | | | | |
| Medium (11-20 schools) | 5.09** | 2.05, 8.14 | 0.135 | -0.67, 4.96 |
| Large (21-30 schools) | 6.55** | 1.91, 11.18 | 3.93 | -0.32, 8.19 |
| Very Large (≥ 31 schools) | 18.49*** | 13.12, 23.87 | 8.92*** | 4.81, 13.04 |
| Locale (ref Urban) | | | | |
| Suburban | -23.27*** | -27.51, -19.03 | -10.87*** | -14.79 -6.94 |
| Town | -18.36*** | -22.47, -14.25 | 1.98 | -1.06, 5.02 |
| Rural | -18.07*** | -21.91, -14.22 | 7.41*** | 4.51, 10.32 |
| Social Deprivation Index Score (25-point change) | 14.88*** | 13.68, 16.07 | 5.99*** | 4.89, 7.09 |
| State Medicaid Expansion Status (ref Not Expanded) | | | | |
| Expanded | 5.63* | 1.10, 10.16 | 13.28*** | 9.53, 17.03 |
| Region (ref Western) | | | | |
| Mountain Plains | -8.63 | -17.64, 0.39 | 2.70 | -6.18, 11.57 |
| Southwest | -0.20 | -7.64, 7.23 | 3.05 | -2.28, 8.39 |
| Midwest | 3.65 | -5.43, 12.73 | 4.30 | -1.34, 9.93 |
| Mid-Atlantic | 8.30 | -1.43, 18.02 | 5.52 | -0.98, 12.03 |
| Northeast | 14.51*** | 7.34, 21.68 | 8.07** | 3.28, 12.87 |
| Southeast | 12.74** | 4.09, 21.39 | 13.64** | 5.87, 21.40 |

Table shows marginal effects in terms of prevalence of CEP participation by school and area characteristics, relative to base category, adjusted for covariates for 53,391 schools eligible for CEP between 2014-2020. Results are displayed in percentage points. Results of joint tests for all categorical variables that all categories are equal to zero were statistically significant at the p<.001 level.

^a^Unadjusted models were separate logistic regression models, adjusted for year, and with standard errors accounting for clustering at the district level.

***p<.001 **p<.01 *p<.05

**Table S3.** Relative associations (marginal effects) of school and area characteristics with prevalence of participation in the Community Eligibility Provision, stratified by whether schools were in a partially adopting school district

|  | **Schools in Partially Adopting Districts (N=27,142)** | | **Schools Not in Partially Adopting Districts (N=20,569)** | |
| --- | --- | --- | --- | --- |
|  | **Adjusted Average Marginal Effect** | **95% Confidence Interval** | **Adjusted Average Marginal Effect** | **95% Confidence Interval** |
| **School-Level Characteristics** | | | | |
| Level (ref Elementary) | | | | |
| Middle/Jr. High | -5.22*** | -7.50, -2.95 | 3.03*** | 1.85, 4.22 |
| High | -12.01*** | -15.49, -8.54 | 4.72*** | 3.10, 6.34 |
| Other (e.g. K-12) | -19.54*** | -23.16, -15.91 | -7.71*** | -9.95, -5.46 |
| Enrollment (ref Small: < 400 students)) | | | | |
| Medium (400-699 students) | 2.83** | 1.06, 4.61 | 0.85 | -0.63, 2.32 |
| Large (700-999 students) | 2.76 | -0.40, 5.92 | 1.79 | -0.75, 4.33 |
| Very Large (≥ 1000 students) | 2.44 | -1.81, 6.69 | 1.36 | -2.13, 4.86 |
| ISP Category (ref < 40%) | | | | |
| 40-50% | 7.01*** | 3.97, 10.04 | 4.73*** | 3.41, 6.05 |
| 50-60% | 22.18*** | 18.32, 26.03 | 17.51*** | 15.66, 19.37 |
| 60-70% | 30.80*** | 26.25, 35.35 | 31.59*** | 28.88, 34.29 |
| 70-80% | 30.43*** | 24.81, 36.06 | 37.42*** | 33.02, 41.82 |
| 80-90% | 32.49*** | 25.32, 39.67 | 40.48*** | 34.65, 46.31 |
| 90-100% | 26.92*** | 18.45, 35.39 | 34.36*** | 28.04, 40.69 |
| Race and Ethnicity (25-percentage point change) | | | | |
| Percent Hispanic | 0.04 | -3.30, 3.39 | -4.86*** | -6.80, -2.92 |
| Percent White | -2.42 | -5.65, 0.81 | -6.51*** | -8.38, -4.64 |
| Percent Black | 2.01 | -1.15, 5.18 | -1.46 | -3.62, 0.71 |
| **Area-Level Characteristics** | | | | |
| School District Size (ref Small: ≤ 10 schools) | | | | |
| Medium (11-20 schools) | -0.10 | -3.71, 3.51 | -6.17** | -10.18, -2.17 |
| Large (21-30 schools) | -3.32 | -8.52, 1.87 | -4.11 | -11.62, 3.40 |
| Very Large (≥ 31 schools) | 2.64 | -1.73, 7.00 | -14.01*** | -22.85, -6.17 |
| Locale (ref Urban) | | | | |
| Suburban | -13.64*** | -19.51, -7.77 | -6.17* | -11.93, -1.62 |
| Town | 1.33 | -3.00, 5.66 | 4.11 | -11.62, 3.40 |
| Rural | 5.48** | 1.47, 9.49 | 11.81*** | 7.38, 16.25 |
| Social Deprivation Index Score (25-point change) | 3.99*** | 2.40, 5.58 | 8.13*** | 6.78, 9.49 |
| State Medicaid Expansion Status (ref Not Expanded) | | | | |
| Expanded | 12.48*** | 6.76, 18.19 | 18.62*** | 14.56, 22.68 |
| Region (ref Western) | | | | |
| Mountain Plains | 6.92 | -5.98, 19.82 | 15.02** | 6.21, 23.84 |
| Southwest | 6.58 | -1.50, 14.65 | 9.22*** | 4.33, 14.12 |
| Midwest | 9.50** | 1.73, 17.27 | 4.87 | 0.002, 9.74 |
| Mid-Atlantic | 11.79** | 1.27, 22.31 | 13.26*** | 6.25, 20.27 |
| Northeast | 2.79 | -3.31, 8.90 | 22.02*** | 14.21, 29.82 |
| Southeast | 10.60 | -0.66, 21.85 | 25.95*** | 18.67, 33.24 |

Sample excluded schools in districts with only one school. The sample included a total of 47,711 schools; 27,142 schools were in school districts that partially adopted CEP (mean district size = 83 schools). Among schools in districts that did not partially adopt CEP (N=20,569), 8,735 schools were in districts that fully adopted CEP (mean district size = 16 schools), and 11,834 schools were in districts in which no schools adopted CEP (mean district size = 13 schools).

***p<.001 **p<.01 *p<.05

**Figure S1.** Adjusted associations of school-level characteristics with participation in the Community Eligibility Provision over time (modeled with year as a categorical variable)

Figures show annual prevalence of CEP participation for 53,391 schools eligible for CEP between 2014-2020, by year (treated as a disjoint indicator variable) and category, adjusted for covariates. Standard errors were clustered at the district level. ISP is the school identified student percentage. For school level, “other” represents a combination of primary and secondary school grades not captured by elementary, middle, or high (e.g. K-12). Prevalence by school race and ethnicity demographics were computed for a hypothetical school with 75% Black, Hispanic, or White students, respectively.

**Figure S2:** Adjusted associations of area-level characteristics with participation in the Community Eligibility Provision over time (modeled with year as a categorical variable)

Figures show annual prevalence of CEP participation for 53,391 schools eligible for CEP between 2014-2020, by year (treated as a disjoint indicator variable) and category, adjusted for covariates. Standard errors were clustered at the district level. For SDI, the 25^th^ percentile corresponds to a score of 52/100, 50^th^ percentile to a score of 72/100, and 75^th^ percentile to a score of 87/100.

**Table S4.** Adjusted annual change in log-odds of participation in the Community Eligibility Provision by school and area characteristics, 2014-2020

|  | **Adjusted Annual Change in Log-Odds** | **95% Confidence Interval** |
| --- | --- | --- |
| **School-Level Characteristics** | | |
| Level (ref Elementary) | | |
| Middle/Jr. High | -0.01 | -0.03, 0.01 |
| High | 0.04* | 0.01, 0.06 |
| Other (e.g. K-12) | 0.05** | 0.02, 0.08 |
| Enrollment (ref Small: < 400 students)) | | |
| Medium (400-699 students) | 0.02* | 0.004, 0.04 |
| Large (700-999 students) | 0.02 | -0.01, 0.05 |
| Very Large (≥ 1000 students) | -0.03 | -0.07, 0.02 |
| ISP Category (ref < 40%) | | |
| 40-50% | 0.03 | -0.04, 0.11 |
| 50-60% | <0.001 | -0.07, 0.07 |
| 60-70% | -0.01 | -0.08, 0.05 |
| 70-80% | 0.01 | -0.06, 0.07 |
| 80-90% | 0.02 | -0.06, 0.10 |
| 90-100% | -0.04 | -0.14, 0.05 |
| Race and Ethnicity 1 unit change in log-odds | | |
| Percent Hispanic | <-0.001 | -0.001, 0.001 |
| Percent White | 0.001 | <-0.001, 0.002 |
| Percent Black | 0.001 | <-0.001, 0.002 |
| **Area-Level Characteristics** | | |
| School District Size (ref Small: ≤ 10 schools) | | |
| Medium (11-20 schools) | 0.01 | -0.03, 0.05 |
| Large (21-30 schools) | 0.07* | 0.01, 0.14 |
| Very Large (≥ 31 schools) | 0.01 | -0.04, 0.06 |
| Locale (ref Urban) | | |
| Suburban | 0.02 | -0.02, 0.07 |
| Town | 0.02 | -0.03, 0.06 |
| Rural | 0.01 | -0.04, 0.05 |
| Social Deprivation Index Score 1 unit change in log-odds | <0.001 | -0.001, 0.001 |
| State Medicaid Expansion Status (ref Not Expanded) | | |
| Expanded | -0.03 | -0.07, 0.02 |
| Region (ref Western) | | |
| Southwest | 0.07* | 0.001, 0.13 |
| Mountain Plains | -0.03 | -0.11, 0.06 |
| Midwest | -0.07 | -0.15, 0.01 |
| Southeast | -0.02 | -0.10, 0.05 |
| Mid-Atlantic | -0.04 | -0.12, 0.05 |
| Northeast | 0.47*** | 0.37, 0.57 |

Table shows estimates of average annual change in prevalence of CEP participation between 2014-2020 by school and area characteristics, relative to base category, adjusted for covariates for 53,391 schools eligible for CEP between 2014-2020. Standard errors were clustered at the district level. Results are displayed in terms of log-odds.

***p<.001 **p<.01 *p<.05

References:

1.	National Center for Education Statistics. Common Core of Data. Accessed December 29, 2023. https://nces.ed.gov/ccd/

2.	Food Research Action Center. Community Eligibility. Accessed August 15, 2024. https://frac.org/community-eligibility

3.	Ruffini K. Universal Access to Free School Meals and Student Achievement: Evidence from the Community Eligibility Provision. *J Human Resources*. 2022;57(3):776-820. doi:10.3368/jhr.57.3.0518-9509R3
